# Searching for Novel Cdk5 Substrates in Brain by Comparative Phosphoproteomics of Wild Type and Cdk5^−/−^ Mice

**DOI:** 10.1371/journal.pone.0090363

**Published:** 2014-03-21

**Authors:** Erick Contreras-Vallejos, Elías Utreras, Daniel A. Bórquez, Michaela Prochazkova, Anita Terse, Howard Jaffe, Andrea Toledo, Cristina Arruti, Harish C. Pant, Ashok B. Kulkarni, Christian González-Billault

**Affiliations:** 1 Laboratory of Cellular and Neuronal Dynamics, Department of Biology, Faculty of Sciences, Universidad de Chile, Santiago, Chile; 2 Functional Genomics Section, National Institute of Dental and Craniofacial Research, National Institutes of Health, Bethesda MD, USA; 3 Protein and Peptide Facility, National Institute of Neurological Disorders and Stroke, National Institutes of Health, Bethesda MD, USA; 4 Laboratorio de Cultivo de Tejidos, Sección Biología Celular, Departamento de Biología Celular y Molecular, Facultad de Ciencias, Universidad de la República, Montevideo, Uruguay; 5 Laboratory of Neurochemistry, National Institute of Neurological Disorders and Stroke, National Institutes of Health, Bethesda MD, USA; McGill University Department of Neurology and Neurosurgery, Canada

## Abstract

Protein phosphorylation is the most common post-translational modification that regulates several pivotal functions in cells. Cyclin-dependent kinase 5 (Cdk5) is a proline-directed serine/threonine kinase which is mostly active in the nervous system. It regulates several biological processes such as neuronal migration, cytoskeletal dynamics, axonal guidance and synaptic plasticity among others. In search for novel substrates of Cdk5 in the brain we performed quantitative phosphoproteomics analysis, isolating phosphoproteins from whole brain derived from E18.5 Cdk5^+/+^ and Cdk5^−/−^ embryos, using an Immobilized Metal-Ion Affinity Chromatography (IMAC), which specifically binds to phosphorylated proteins. The isolated phosphoproteins were eluted and isotopically labeled for relative and absolute quantitation (iTRAQ) and mass spectrometry identification. We found 40 proteins that showed decreased phosphorylation at Cdk5^−/−^ brains. In addition, out of these 40 hypophosphorylated proteins we characterized two proteins, :MARCKS (Myristoylated Alanine-Rich protein Kinase C substrate) and Grin1 (G protein regulated inducer of neurite outgrowth 1). MARCKS is known to be phosphorylated by Cdk5 in chick neural cells while Grin1 has not been reported to be phosphorylated by Cdk5. When these proteins were overexpressed in N2A neuroblastoma cell line along with p35, serine phosphorylation in their Cdk5 motifs was found to be increased. In contrast, treatments with roscovitine, the Cdk5 inhibitor, resulted in an opposite effect on serine phosphorylation in N2A cells and primary hippocampal neurons transfected with MARCKS. In summary, the results presented here identify Grin 1 as novel Cdk5 substrate and confirm previously identified MARCKS as a a *bona fide* Cdk5 substrate.

## Introduction

The complexity of cell functions derives not only from the particular content of individual proteins at a given time, but also from their post-translational modifications. The most common post-translational modification of proteins is the phosphorylation of serine, threonine, and tyrosine residues. This is particularly important to cellular mechanisms that integrate extracellular and intracellular signals through selective phosphorylation of proteins leading to specific functional outcomes. Protein phosphorylation plays a key role in many cellular functions such as cell signaling, apoptosis, cell migration, cytoskeletal dynamics and brain development. Cyclin-dependent kinase 5 (Cdk5) is an atypical member of the cyclin-dependent kinase family of which most of the members are key regulators of the cell cycle [1], [2]. Although Cdk5 is ubiquitously expressed, it is mainly active in post-mitotic neurons, where it is activated by neuron-specific activators p35 and p39 [3], [4]. Cdk5 belongs to the proline-directed serine/threonine kinase group, and it phosphorylates many proteins possessing a canonical consensus sequence (S/T)PX(K/H/R) or at least a minimal consensus sequence (S/T)P [5]. Cdk5 is known to phosphorylate cytoskeletal proteins, signaling molecules, ion channels and regulatory proteins that participate in the normal function of the brain and also during neurodegenerative disorders [1], [2], [4], [6]. A detailed analysis of Cdk5^−/−^ mice, which display perinatal lethality and extensive neuronal migration defects, revealed that Cdk5 serves as a key regulator of neuronal migration, neurite outgrowth, and axonal path finding and dendrite development [7], [8].

Given that a large number of the key cellular processes involve Cdk5 activity, suggesting that with the advent of new proteomic techniques many more Cdk5 substrates will be discovered. Interestingly, in the last few years many reports have indicated that Cdk5 also has extra-neuronal functions, such as regulating gene transcription, vesicular transport, apoptosis, cell adhesion, and migration in many cell types and tissues [2], [9]. In the past, most of the Cdk5 substrates were discovered by classical biochemical and molecular approaches. However, there have been some efforts to identify Cdk5 substrates using high throughput screening (HTS). For example, Gillardon and colleagues used two-dimensional electrophoresis and mass spectrometry to study the protein phosphorylation patterns in cultured rat cerebellar granule neurons treated with Indolinone A, a Cdk5 inhibitor. Although these researchers did not find any specific substrates directly phosphorylated by Cdk5, their study demonstrated the changes in the phosphorylation status of certain proteins. Some results suggest that inhibiting Cdk5 activates stress proteins that may protect neurons against subsequent injurious stimuli [10]. Gillardon and colleagues also analyzed global changes in protein phosphorylation and gene expression in cultured cerebellar granule neurons by (^32^P) orthophosphate labeling after the administration of a Cdk5 inhibitor. They found that Indolinone A treatment regulated protein phosphorylation and gene expression of candidates involved in neuronal survival, neurite outgrowth, and synaptic functions [11]. More recently, using high-density protein microarrays of Indolinone A-treated cerebellar granule neurons the same group identified two other potential Cdk5 substrates: Protein phosphatase 1 regulatory subunit 14A and Coiled-coil domain containing protein 97 [12]. Another phosphoproteomic study using a solid-phase capture-release-tag approach identified and suggested that Tau and MAP2 contain a Cdk5 phosphorylation motif [13].

In this study, we carried out systematic phosphoproteomic analysis of the Cdk5^+/+^ and Cdk5^−/−^ mice brains. We analyzed E18.5 mouse brain lysates using IMAC followed by iTRAQ and mass spectrometry. We discovered forty proteins with decreased phosphorylation at Cdk5 motifs in the Cdk5^−/−^ brain. These proteins contained one or more phosphopeptides containing the canonical consensus phosphorylation motif for Cdk5 [5]. From the list of 40 protein candidates derived from HTS, we selected two proteins related to neurite outgrowth for further analysis. They were MARCKS (Myristoylated Alanine-Rich C Kinase Substrate) and Grin1 (G-protein-regulated inducer of neurite outgrowth). MARCKS was originally discovered as the main PKC substrate in the CNS [14], [15], but it is also phosphorylatable by proline-directed kinases [16]. Interestingly for the purpose of this study is the fact that Cdk5 phosphorylates MARCKS at Ser25 in chick neuroblasts *in vivo* (Ser27 in mammals) [29]. Grin1 is highly expressed in the nervous system during development and it has been suggested that it has an important function in neuronal migration and brain formation [17]. The results presented here confirmed that chick MARCKS is phosphorylated by murine endogenous Cdk5 when it, was overexpressed in neuroblastoma N2a cells, altogether with p35. For the first time, we also show that Grin1 is a substrate for this kinase in analogous experimental conditions. Both proteins were not phosphorylated when Cdk5 was inhibited by roscovitine.

## Materials and Methods

### Animals

Cdk5 knockout mice (Cdk5^−/−^) were generated as previously described [7]. Female Cdk5^+/−^ mice pregnant were euthanized at E18.5 with a lethal injection of Ketamine/Xilacine mix. The genotype of each mouse was determined by PCR from DNA obtained from tail biopsies. For primary culture of rat hippocampal neurons, female rats Sprague-Dawley pregnant were euthanized at E18.5 with a lethal injection of Ketamine/Xilacine mix. These studies were performed in compliance with the National Institutes of Health's Guidelines on the Care and Use of Laboratory and Experimental Animals. All experimental procedures were approved by the Animal Care and Use Committee of the National Institute of Dental and Craniofacial Research, NIH, and the Bioethical Committee of the Faculty of Sciences, University of Chile, according to the ethical rules of the Biosafety Policy Manual of the National Council for Scientific and Technological Development (FONDECYT).

### Phosphoprotein enrichment

Ten whole brains of E18.5 from Cdk5^+/+^ and Cdk5^−/−^ mice were homogenized using the buffer provided in the PhosphoProtein Purification Kit (QIAGEN #37101, Valencia, CA, USA), in accordance with the manufacturer's protocol. Briefly, protein extracts were loaded in columns, washed, eluted, and quantified by the Bradford method [18].

### Protein digestion

One mg of phosphoprotein obtained from brain lysates was dried in the SpeedVac (ThermoSavant, Farmingham, NY, USA) and then dissolved in 8 M urea, 0.4 M NH_4_HCO_3_ for reduction by dithiothreitol and alkylation by indole acetic acid. After dilution into 2 M urea, 0.1 M NH_4_HCO_3_, tryptic digestion was performed as described before [19].

### Quantitative proteomics (iTRAQ) of phosphoproteins

The identification and quantitation of phosphoproteins obtained from Cdk5^+/+^ and Cdk5^−/−^ mice were performed as previously described [20]. Briefly, we performed an iTRAQ procedure which is a non-gel based technique that incorporates isotope-coded covalent labeling of the N-terminus and side-chain amines of peptides with tags of varying mass (114.1; 115.1; 116.1; 117.1) (Absciex, Foster City, CA, USA). The samples were combined and analyzed by tandem mass spectrometry (MS/MS). In order to identify the labeled peptides and corresponding proteins, we used SEQUEST (http://fields.scripps.edu/sequest/) to quantify the low molecular mass reporter ion generated by the fragmentation of the attached tag and consequently the peptides and proteins from which they originated.

### LC/MS/MS analysis

Samples were analyzed by LC/MS/MS on LTQ XL (linear trap quadraplole) with 2 Surveyor MS Pump plus HPLC pumps and Micro AS (Thermo Scientific, Waltham, MA, USA) and they were equipped with an Advance ESI (electrospray ionization) source (Michrom Bioresources Inc., Auburn, CA, USA). The equipment was used with an instrument configuration, columns, gradient, and source conditions as previously described [19]. The LTQ XL was set up to acquire a survey scan between *m/z* 400–1400 followed by a PQD MS/MS spectrum on each of the top 10 most abundant ions in the survey scan. Source conditions were as previously described and are listed here as follows: capillary temperature, 165°C; sheath gas flow, 2 U; spray voltage, 1.6 kV. Key optimized PQD instrument parameters (32) were as follows: CE, 35%; isolation width, 3 *m/z*; activation Q, 0.700; activation time, 0.100 ms; minimum signal threshold, 10,000 cts; dynamic exclusion, repeat count 2, repeat duration 30 s, exclusion duration 60 s; MS/MS target, 4.0 X *e* ^4^; maximum fill time, 100 ms; 4 microscans.

### Data analysis and iTRAQ quantitation

PQD MS/MS spectra were searched in the mouse database utilizing BioWorks 3.3.1 SP1 (Thermo Scientific) for site-specific phosphopeptide identification and iTRAQ quantification. The search parameters were set and data analysis was done as previously described [19]. Briefly, the search parameters were set as follows: static modifications: C = 57.0215, N-term = 144, and K = 144; differential modifications: S, T, Y = 79.9799 and M = 16. The search results were reported in descending order of the X correlation (XC) score subject to the default charge *vs.* XC filter: 1^+^ = 1.50, 2^+^ = 2.00, and 3^+^ = 2.50. Sequence assignments including the specific phosphorylated residue, were based on the selection of the phosphopeptide with the highest XC score, which is concurrent with the second ranked peptide displaying a ΔCn (the difference in the normalized XC score between the top scoring sequence and the next highest scoring sequence) of 0.1. MS/MS spectra were manually reviewed for spectral quality and the assignment of most major ions.

### Transient transfection of N2A cell line

Mouse neuroblastoma N2A cells (ATCC#CCL-131) were transiently co-transfected with pBC12/pCMV-60k plasmid (chicken MARCKS) from Dr. Perry Blackshear [21] and pBI-p35/EGFP vector [22] and tTA vector in Opti-MEM with Lipofectamin 2000 (Invitrogen, Carlsbad, CA, USA) in DMEM. After 4 h of transfection, the solution was changed for DMEM plus 10% FBS, and the cells were treated with roscovitine (20 µM) during 20 h until protein extracts were made.

### Transient transfection of primary hippocampal neurons in culture

Rat primary hippocampal neurons were performed as described before [23]. Briefly, hippocampal neurons from Sprague-Dawley rat embryos (E18.5) were dissociated after treatment for 20 min with 0.25% (w/v) trypsin (Gibco). 3×10^5^ cells were plated in coverslips previously coated with poly-D-lysine 1 mg/ml (Sigma-Aldrich) in Neurobasal medium (Gibco) including 10% horse serum and Glutamine (Gibco). Then, neurons were transiently transfected with 0.6 µg of plasmid DNA (0.3 µg of CMV-GFP vector and 0.3 µg of chicken MARKCS vector) for 2 h and then the medium was replaced with Neurobasal medium supplemented with B27 and Glutamax (Gibco) in the absence of serum. Cells were kept in a humidified atmosphere of 5% CO2 at 37°C during 48 h and half of the covers were treated with roscovitine 10 µM. After 24 h neurons were fixed for posterior immunofluorescence against phosphor Ser25 MARCKS and DAPI.

### Western Blot

Protein concentration was determined by the Bradford protein assay [18]. In brief, 30 µg of protein were loaded and run in 10% or 12% polyacrylamide gels at 100 V. The proteins were then transferred to nitrocellulose membranes in 120 mM glycine, 125 mM Tris, 0.1% SDS and 20% methanol, at 200 mA for 1 h. Then, the nitrocellulose membranes were blocked in 5% non-fat dry milk in PBS1X-0.1% Tween-20 for 1 h at room temperature and incubated overnight with primary antibodies diluted in 5% non-fat dry milk in PBS1X-0.1% Tween-20 at 4°C. The nitrocellulose membranes were incubated with the following antibodies: mAb 3C3 (mouse monoclonal anti-S25p-MARCKS [24]) diluted in blocking solution; Polo52 (rabbit polyclonal anti-MARCKS antibody, serum diluted 1∶2000 [25]); anti-β-actin 1∶2000 (sc-69879, Santa Cruz Biotechnology); anti-p35 (sc-820, Santa Cruz Biotechnology); α-tubulin (T9026) and phospho-serine (P5447) antibodies were obtained from Sigma (St. Louis, MO, USA); Phospho-MAPK/CDK Substrates Rabbit mAb #2325 (Cell Signaling Technology, Denver, MA, USA); anti-Grin1 116 was obtained from Dr. Tohru Kozasa from Department of Pharmacology, College of Medicine, University of Illinois, Chicago USA. Secondary antibodies horseradish peroxidase-conjugated goat anti-mouse and anti-rabbit antibodies were obtained from Jackson ImmunoResearch (West Grove, PA, USA). Secondary antibody goat anti-mouse IgG-HRP (31430, Thermo Scientific) used at a dilution of 1∶10000 in blocking solution. Nitrocellulose membranes were washed three times with PBS containing 0.1% Tween-20 for 15 min and then the labeling was visualized with ECL reagent (32106, Thermo Scientific). All of Western blot data are representative of at least three independent experiments.

### Immunoprecipitation assays

500 µg of protein from the whole mouse brain or the transfected N2A cells were immunoprecipitated in 500 µl of TPER buffer (Pierce) with 1–2 µg of Grin1, MARCKS, or Cdk5 antibodies overnight at 4°C in a shaker. Then, 30 µl of Protein A/G (Sigma) was added and incubated for 4 h at 4°C in a shaker. The immunocomplexes were centrifuged at 4°C during 5 min at 1000×g and washed 4 times in cold PBS1X. The loading buffer 5× (tris-HCl 62 mM, Glycerol 25%, SDS 2%, β-mercaptoethanol 10%, bromophenol blue 0.1%) was added into the beads and samples, and they were boiled at 95°C for 5 min and ran in SDS-PAGE gels.

### Gene ontology analysis

The PANTHER (Protein analysis through evolutionary relationships) Classification System was used to classify proteins. The proteins were classified according to family and subfamily of molecular function, biological process and signaling pathway [26], [27]. The Visualization and Integrated Discovery (DAVID) software was also used to analyze the data [28].

### Statistical Analysis

All experiments were performed at a minimum of three times. A statistical evaluation was performed with GraphPad Prism software, version 5.0 (GraphPad, San Diego, CA). The significant differences between experiments were assessed by an unpaired Student test where α was set to 0.05.

## Results

### Phosphoproteomic analysis of Cdk5^−/−^ brain

The proteomic analysis of iTRAQ-labeled tryptic peptides from control and Cdk5 null brains revealed 78 non-redundant phosphorylated sites that were decreased in Cdk5^−/−^ brain. The majority of these phosphosites (55/78, 70%) present in 40 phosphoproteins have a minimal consensus Cdk5 motif of (S/T)P (Table 1). However, a decreased phosphorylation at these sites may also reflect the indirect effects of other proline-directed kinases regulated by Cdk5. Similar indirect effects have been reported for the kinases in the MEK-ERK [29] or JNK [30] pathways. In order to further refine identification of potential sites directly phosphorylated by Cdk5, we performed bioinformatics analysis using two sequence-based prediction tools for Cdk5 substrates: a position specific scoring matrix (PSSM) [5] and a web tool based in artificial neural networks (ANN), called NetPhosK [31]. The scores calculated by both tools are shown in Table 1. Although PSSM allows for better accuracy in predictions [5], NetPhosK allows a comparative study, with a parallel scoring for 16 other kinases. Interestingly, for 27 sites in 20 phosphoproteins (49%), Cdk5 is the most likely kinase that phosphorylates these sites (Table 1 asterisks). Two of these potential direct Cdk5 substrates, corresponding to Ser27 of MARCKS and Ser369 of Grin1, were selected for further analysis.

**Table 1:** Putative Cdk5-dependent phosphorylation sites downregulated in Cdk5^−/−^ brains.

Name	Gene	Site	% of decrease	PSSM score	NetphosK score
40S ribosomal protein S3	Rps3	**T221** b ^,^ c	97.4	1.1205	0.53
Abl interactor 1	Abi1	**S183**	76.7	0.9482	0.59
Alpha-adducin	Add1	**S12**	92.7	0.7623	0.57*
APC membrane recruitment protein 2	Amer2	S589	100	-	0.50
Bcl-2-associated transcription factor 1	Bclaf1	**S510**	100	0.8775	0.51
		**S656**	100	-	0.56*
Calmodulin-regulated spectrin-associated protein 1	Camsap1	**S1069** c	100	1.2446	0.54
Calmodulin-regulated spectrin-associated protein 3	Camsap3	**S368**	100	0.7122	0.66*
Clathrin coat assembly protein AP180	Snap91	**S300**	92.5	0.7577	0.69*
Collapsin response mediator protein 1	Dpysl1	**T509** a ^,^ c	57.2	0.5071	0.49
Collapsin response mediator protein 2	Dpysl2	**T509** a	78.1	0.7100	0.40
Collapsin response mediator protein 4	Dpysl3	**T509** a ^,^ c	100	0.7929	0.48
DNA ligase 1	Lig1	S51^c^	100	0.7076	0.60*
E3 ubiquitin-protein ligase TRIM2	Trim2	**S428**	91.1	0.7835	0.68*
*G protein-regulated inducer of neurite outgrowth 1*	*Gprin1*	***S369***	*74.3*	*0.8067*	0.68*
		**S691**	100	0.6710	0.44
Growth-associated protein 43	Gap43	**S96**	34.5	0.9276	0.36
		**T172**	100	0.8226	0.43
Hepatoma-derived growth factor	Hdgf	**S165**	75	0.8228	0.44
Heterogeneous nuclear ribonucleoprotein D0	Hnrnpd	**S83**	37.1	-	0.52*
Kinesin light chain 1	Klc1	**S459**	100	1.0709	0.39
MARCKS-related protein	Marcksl1	**S22**	84.3	1.1036	0.43
Microtubule-associated protein 1B	Map1b	**T527**	100	1.0861	0.47
		**S1307**	46.6	1.0589	0.35
		**S1438**	47.4	1.1018	0.53*
Microtubule-associated protein 2	Map2	**T1650**	87.9	-	0.69*
Microtubule-associated protein tau	Mapt	**T468**	30	0.7591	0.51*
		**S470**	42.4	0.9090	0.56*
		**T473** a	30	0.7174	0.68*
		**T504** a ^,^ b	46.6	0.6513	0.70*
		**T523** a	93.2	0.7551	0.73*
		**S527** a ^,^ b	97.6	0.5220	0.68*
*Myristoylated alanine-rich C-kinase substrate*	*Marcks*	***S27*** c	*80.7*	*0.9177*	0.53*
		**S138**	32.9	0.8514	0.47
		**T143** c	41.1	0.6899	0.55
Na(+)/H(+) exchange regulatory cofactor NHE-RF1	Slc9a3r1	**S275**	51.1	0.5991	0.60*
Nestin	Nes	**S1837**	91	0.6777	0.64*
Neuronal migration protein doublecortin	Dcx	**T336**	67.8	0.7194	0.73*
		**S339** a	89.3	0.7502	0.74*
Phosphatidylinositol 4-kinase beta	Pi4kb	T517	100	0.9211	0.43
Plakophilin-4	Pkp4	**S509**	100	0.9879	0.48*
Programmed cell death protein 4	Pdcd4	**S94**	100	0.8618	0.58*
Protein SDE2 homolog	Sde2	**S269**	100	0.6656	0.54
Ras GTPase-activating protein-binding protein 1	G3bp1	**S231**	100	0.8606	0.38
Ras GTPase-activating protein-binding protein 2	G3bp2	**T227**	92.5	0.9730	0.37
Rho GTPase-activating protein 1	Arhgap1	**S51**	83.1	-	0.46
RNA-binding protein Raly	Raly	**S135** c	55.7	1.0370	0.48
SAFB-like transcription modulator	Sltm	**S289**	89.9	-	0.37
Serine/threonine-protein kinase DCLK1	Dclk1	**S32**	34.3	-	0.71*
		**S334**	64.8	0.5840	0.66*
Stathmin	Stmn1	**S25** a ^,^ b ^,^ c	78.7	0.4295	0.36
		**S38** a ^,^ b ^,^ c	95.3	1.0112	0.72*
Stathmin-2	Scg10	**S62**	52.4	0.9175	0.48
T-box brain protein 1	Tbr1	**S84**	100	0.8802	0.38
U1 small nuclear ribonucleoprotein 70	Snrnp70	**S226** c	84.9	0.9905	0.57*

In **bold** type phosphorylated sites previously described in other mouse brain phosphoproteomic studies but lacking an assigned protein kinase responsible for such a phosphorylation.

a : phosphorylated by Cdk5,

b : phosphorylated by Cdk1;

c : phosphorylated by Cdk2.

PSSM scores determined as Borquez et al, 2013. NetphosK scores calculated using public protein prediction tool.

Asterisks (*) indicated Cdk5 is the best kinase for a given site.

Candidates validated in the present study are presented in italics type.

### Gene ontology analysis of altered phosphoproteins in Cdk5^−/−^ brain

**Figure 1:**
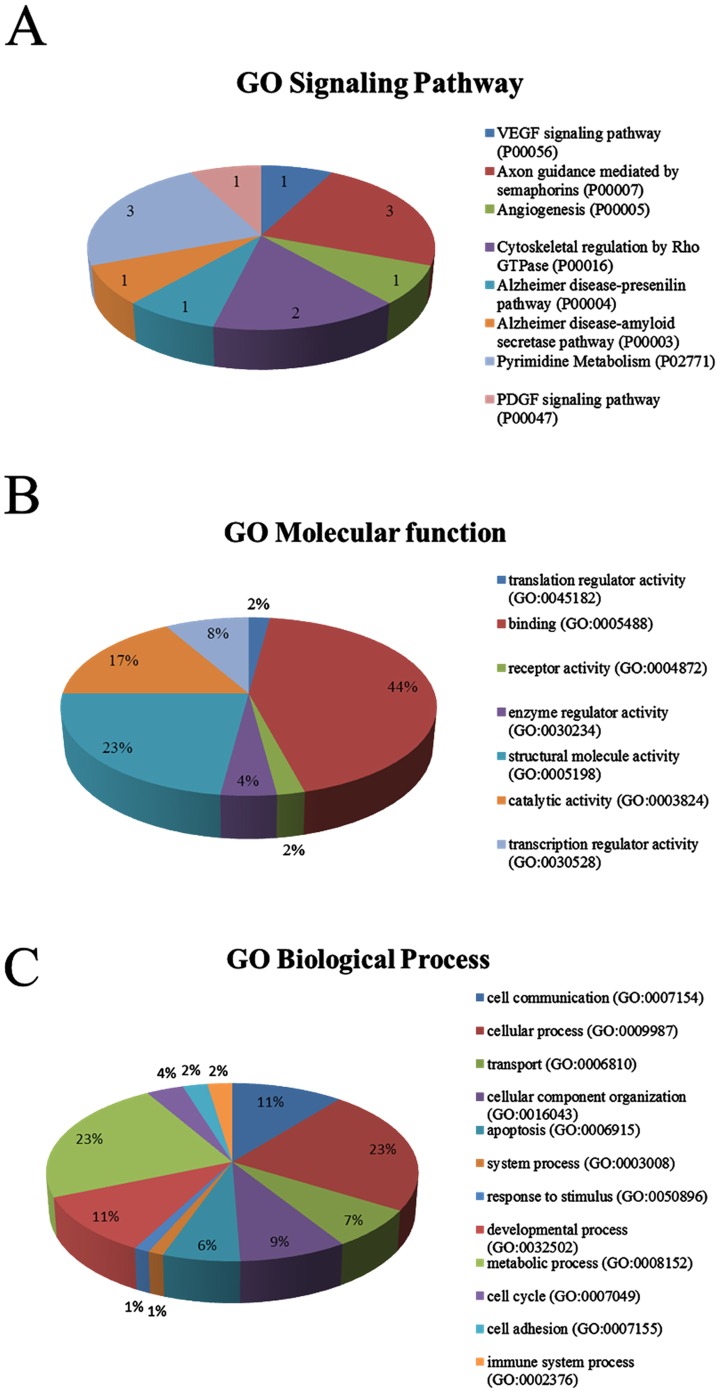
Functional classification of the altered phosphoproteins in Cdk5^−/−^ brain. The phosphorylated proteins identified in our analysis were classified into the following groups: A) Signaling pathways, B) Molecular functions and C) Biological processes. Percentages are expressed as gene hits against total number of process hits. The cellular process is defined as any process that is carried out at the cellular level, but not necessarily restricted to a single cell. The system process is defined as a multicellular organismal process carried out by any of the organs or tissues in an organ system. An organ system is a regularly interacting or interdependent group of organs or tissues that work together to carry out a biological objective. A response to stimulus is defined as any process that results in a change in state or activity of a cell or an organism (in terms of movement, secretion, enzyme production, gene expression, etc.) as a result of a stimulus. The process begins with detection of the stimulus and ends with a change in state or activity or the cell or organism.

In order to get an overview of the altered phosphoprotein profile in Cdk5^−/−^ brains, we classified all of the phosphorylated proteins with the canonical Cdk5 motif which we identified in our experimental analysis in three different groups, namely signaling pathways (Figure 1A), molecular functions (Figure 1B) and biological processes (Figure 1C) according to their annotation in PANTHER. Thirteen of 40 phosphoproteins (30%) were classified in the category of the signaling pathways (Figure 1A). This classification assigned the phosphoproteins to eight different signaling pathways: 1) VEGF signaling (1/13); 2) axon guidance mediated by semaphorins (3/13); 3) angiogenesis (1/13); 4) cytoskeletal regulation by Rho GTPase (2/13); 5) Alzheimer disease-presenilin (1/13); 6) Alzheimer disease-amyloid secretase (1/13); 7) pyrimidine metabolism (3/13); and 8) PDGF signaling (1/13) (Figure 1A). In terms of molecular functions, we identified that 21 of the 40 phosphoproteins that were associated with binding category (GO: 0005488) which is defined as the selective, non-covalent, often stoichiometric, interaction of a molecule with one or more specific sites on another molecule (Figure 1B). These functions correspond to protein binding, receptor binding, cytoskeleton binding and nucleic acid binding. Some of these phosphoproteins belong to more than one binding subcategory. Finally, the 40 phosphoproteins identified in this study were classified into biological processes corresponding to metabolic processes, cell communications, developmental processes and cellular components organization, among others (Figure 1C). Alternatively, by using the bioinformatics tool DAVID, 11 phosphoproteins were clustered into the neuron projection process (GO: 0043005) which is defined as a prolongation or process extending from nerve cells (axon or dendrites) (data not shown).

### Cdk5 and MARCKS phosphorylation at Ser27

**Figure 2:**
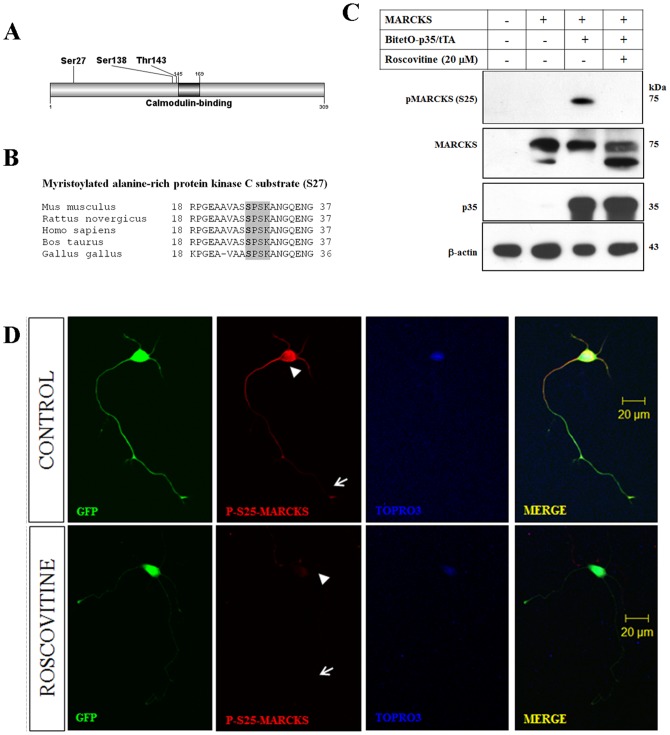
Cdk5 phosphorylation of MARCKS Ser27 in mouse. A) Schematic representation of MARCKS protein with the localization of phosphorylation sites that decreased in our phosphoproteomic study. B) MARCKS protein sequence alignment showing a conserved Cdk5 motif between species. Shaded boxes show conserved amino acids, bold amino acid is the phosphorylation site. C) Western blot analysis of pSer25, MARCKS and p35 in N2A cells co-transfected with MARCKS, MARCKS and pBI-p35/EGFP and MARCKS and pBI-p35/EGFP plus roscovitine. D) Immnunofluorescence of phosphor Ser25 MARCKS in hippocampal neurons transfected with MARCKS and CMV-GFP. Upper panel shows the control condition and lower panel shows roscovitine treated neurons. The expression of Phospho S25 MARCKS was detected on soma (white head arrow) and neurites (white arrow) of control cells. However, the treatment with roscovitine (10 µM during 24 h) decreased MARCKS phosphorylation at both compartments. Nuclei was stained with DAPI.

MARCKS has at least 7 sites that can be phosphorylated by MAPK and/or Cdks [32]. By using phosphoproteomics analysis, we found three phosphorylation sites on MARCKS that significantly decreased in the Cdk5^−/−^ brain: Ser27 (80.7%), Ser138 (32.9%) and Thr143 (44.1%) (Figure 2A). A recent report indicated a decreased phosphorylation of Ser25 on MARCKS in chicken retinal neuroblasts treated with roscovitine or olomuicine (Cdk5 inhibitors), suggesting that Cdk5 was mainly responsible for the phosphorylation of this epitope [25]. Interestingly, MARCKS could be phosphorylated at Thr143 in vitro by Cdk2-cyclin A [33]. Since mouse MARCKS-Ser27 is a homologue residue to chicken MARCKS-Ser25, we expressed chicken MARCKS in mouse N2A neuroblastoma cells. This was to study the potential changes in MARCKS phosphorylation using a specific antibody that recognizes chicken MARCKS phosphorylated at Ser25 (pSer25) (mAb3C3) [24]. Interestingly, chicken MARCKS lacks the serine residue located -1 respect to phosphorylated the serine (Figure 2B). This residue seems to be critical for epitope recognition, because the pSer25 antibody did not recognize rat or mouse MARCKS. We overexpressed chicken MARCKS in neuroblastoma N2A cells along with a tetracycline inducible system for p35 over-expression [22]. We found that the phosphorylation of Ser25 on chicken MARCKS was detected only when we over-expressed p35 (Figure 2C). However, this phosphorylation was undetectable when N2A cells co-transfected with MARCKS and p35 were treated with roscovitine (Figure 2C). Additionally, we evaluated the pharmacological inhibition of Cdk5 in primary hippocampal neurons co-transfected with chicken MARCKS and CMV-GFP vectors. To determine which neurons were transfected we evaluated GFP expressing neurons. pSer25 MARCKS was detected on soma (white head arrow) and neurites (white arrow) of control hippocampal neurons (Figure 2D). However, the treatment with roscovitine (10 µM during 24 h) significantly decreased expression of this phospho-epitope in both compartment suggesting that phosphorylation of Ser25 in MARCKs was dependent on Cdk5 activation (Figure 2D). Our results are supported by an earlier report in which the phosphorylation of chicken MARCKS pSer25 was inhibited by treatment with roscovitine [25]. We then used NetPhosK tool to evaluate which of the three MARCKS sites that were showing decreased phosphorylation in Cdk5 null brains was the best predicted Cdk5 site. As showed in Table 1, amongst Ser27, Ser138 and Thr143 sites, the bioinformatics approach identified Ser27 as a potential Cdk5 substrate the preferred Cdk5 site, confirming results from our phosphoproteomic analysis, heterologous expression and primary neuron assays (Table 1). MARCKS expression had been detected primarily in small dendrites, axons and axon terminal (Table S1). In summary, our results clearly indicate that Cdk5 phosphorylates MARCKS at Ser27 in the mouse brain.

### Cdk5 and Grin1 phosphorylation at Ser369

**Figure 3:**
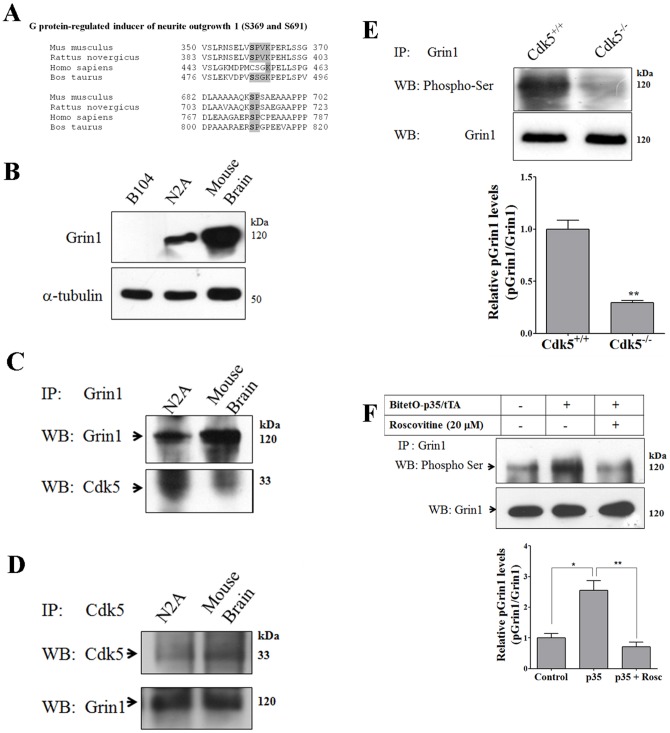
Cdk5 phosphorylates Grin1 at Ser369. A) Grin1 alignment showing Ser369 and Ser691 sequence as Cdk5 motifs. The shaded boxes show conserved amino acids, bold amino acid is the phosphorylation site. B) Detection of Grin1 in rat B104 and mouse N2A neuroblastoma cells and mouse brain. C) Immunoprecipitation of Grin1 and Western blot detection of Cdk5 and Grin1 in N2A cells and mouse brain. D) Immunoprecipitation of Cdk5 and Western blot detection of Grin1 and Cdk5 in N2A cells and mouse brain. E) Immunoprecipitation of Grin1 and Western blot detection of Grin1 and phospho Serine (using an antibody that recognize phosphorylation on SPXK) brain of Cdk5^+/+^ and Cdk5^−/−^ mice. F) Immunoprecipitation of Grin1 and Western blot detection of phospho serine and Grin1 in N2A cells transfected with pBI-p35/EGFP and tTA, and pBI-p35/EGFP and tTA plus roscovitine. All data are presented as mean and SEM (*n* = 3). * p<0.05, ** p<0.01 (t-Test).

Grin1 protein is highly expressed in the developing brain, whereas its expression is more restricted in adult stage [17]. It is highly enriched in growth cones, suggesting that it may be neuron-specific (Table S1). Here, by conducting phosphoproteomics analysis, we found that Grin1 displays two phosphorylation sites Ser369 (74.3% decrease) and Ser691 (100% decrease) that were significantly decreased in Cdk5^−/−^ brain. Ser369 corresponded to a classical consensus sequence for Cdk5, which is conserved in mice and rats but not in humans (Figure 3A), while Ser691 is a KSP motif, which resembles the consensus site for Cdk5 phosphorylation in neurofilaments [34]. To confirm our phosphoproteomic analysis, we analyzed the expression of Grin1 in rat (B104) and mouse (N2A) neuroblastoma cells and the mouse brain. Grin1 antibody only recognized mouse protein, but it did not recognize rat protein (Figure 3B). In addition, we immunoprecipitated Grin1 from N2A cells and the mouse brain and we detected Cdk5 by Western blot (Figure 3C). Similarly we conducted the reciprocal immunoprecipìtation with Cdk5 from N2A cells and the mouse brain and we detected Grin1 by Western blot (Figure 3D). These combined results suggest an interaction between Grin1 and Cdk5. Moreover, the levels of serine phosphorylation in Grin1 were considerably reduced in Cdk5^−/−^ brain as detected with an antibody that recognizes phosphorylated SPXK motif (Figure 3E). This antibody preferentially recognizes Ser369. In addition, we found that the serine phosphorylation of Grin1 increased in N2A cells over-expressing p35, while roscovitine treatment of N2A cells over-expressing p35 had the opposite effect (Figure 3F). Moreover, by using bioinformatics tools NetPhosK we found that the best kinase that phosphorylated Ser369 on Grin1 is Cdk5 (Table 1). Our results confirm phosphoproteomic analysis and indicate that Cdk5 phosphorylates Grin1 at Ser369.

## Discussion

Cdk5, a serine/threonine protein kinase, is involved in many important cellular processes associated with brain development and function. It is also implicated in disease processes associated with neurodegeneration. Cdk5 brings about its effect by phosphorylating a large number of target substrates, resulting in their activation or deactivation. These substrates play key roles in hippocampal neurogenesis, neuronal migration, cytoskeletal dynamics and synaptic plasticity [35]. It is therefore not surprising that novel substrates of Cdk5 that are being identified have important functions in both CNS and PNS [6], [36]. In the present study, we carried out comparative quantitative phosphoproteomic analysis of E18.5 Cdk5^+/+^ and Cdk5^−/−^ brains to search for new Cdk5 substrates. We carried out phosphoprotein enrichment using the IMAC procedure and utilized iTRAQ for peptide labeling and quantification. We found decreased phosphorylation in 55 Cdk5-consensus sites in 40 phosphoproteins from Cdk5^−/−^ brain as compared with Cdk5^+/+^ brains. The phosphoproteins found in our study are involved in different signaling pathways and regulate several cellular processes in the nervous system such as cytoskeletal regulation, axonal growth, axonal guidance and synapse formation. Cdk5 is known to regulate these biological processes through the phosphorylation of key substrates such as PAK1, β-catenin, Src, Nudel, synapsin, MUNC18, and amphyphysin among others proteins [1], [35].

**Figure 4:**
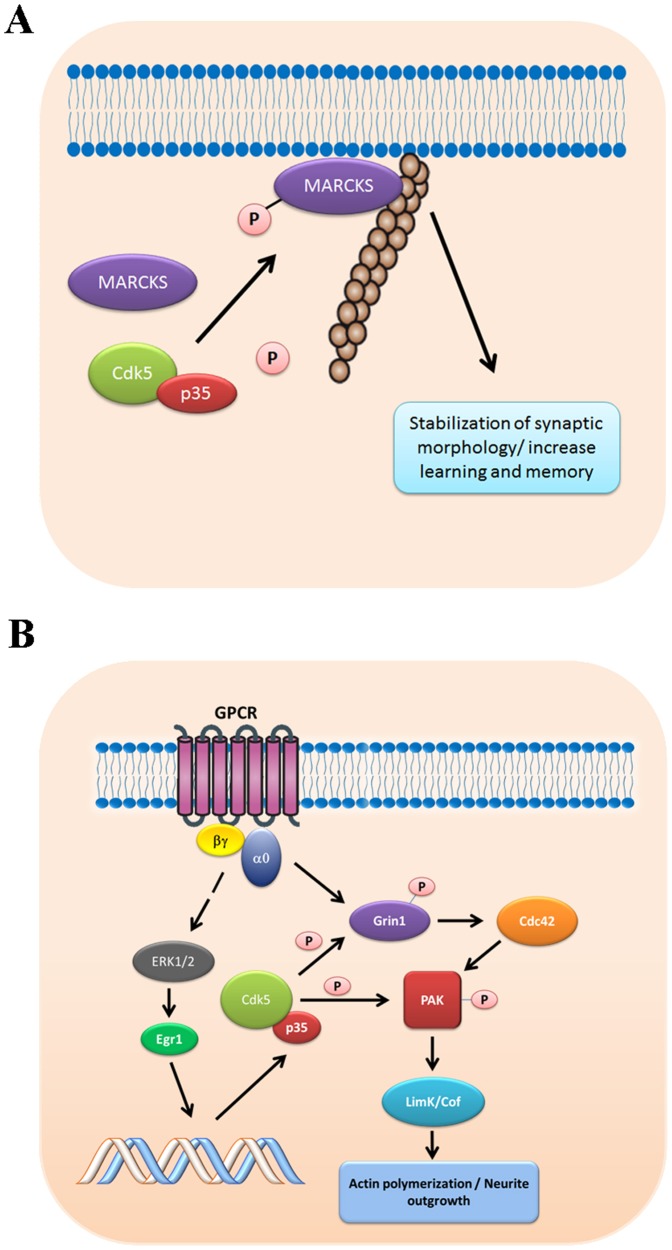
Proposed model illustrating potential roles of phosphorylated Grin1 by Cdk5. A) Phosphorylation of MARCKS by Cdk5 could modulate its interaction with actin filaments leading to stabilization of actin cytoskeleton. B) Involvement of Grin1 phosphorylation by Cdk5 in actin dynamics and neurite outgrowth. GPCR stimulation activates MAPK signaling pathway with increased of Egr1 and p35 expressions and subsequent increases in Cdk5 activity, which in turn phosphorylate Grin1. Additionally, GPCR stimulation promotes neurite outgrowth possibly mediated by the phosphorylation of Grin1 by Cdk5 and Cdc42-PAK-LimK-Cofilin pathway.

MARCKS and Grin1 were two phosphoproteins among the total of 40 identified in our HTS phosphoproteomic analysis of Cdk5^−/−^ brain. We further characterized the Cdk5-mediated phosphorylation of MARCKS and Grin1 in N2A cell line and mouse brain. MARCKS plays a pivotal role during neural development, since the inactivation of MARCKS gene results in abnormal brain development and perinatal death [37]. The functional role of MARCKS has been associated with regulation of growth cone adhesion, axon pathfinding [38] and dendritic spine morphology [39]. Interestingly, the defects found in mice lacking MARCKS were restored by the expression of certain mutated non-phosphorylated form of MARCKS, suggesting that some of the MARCKS functions were independent of its phosphorylation by PKC and possibly involved another phosphorylation sites phosphorylated by other kinase(s) [40]. In support of this suggestion, MARCKS phosphomutants which were used to restore the expression in null mice were indeed in a specific PKC region. MARCKS is also phosphorylated by MAPK and Cdks [32] and its Ser27 is phosphorylated in vitro by the Cdk2-cyclin E complex [41]. The phosphorylation of Ser27 (corresponding to Ser25 in chicken) is dependent on the integrity of actin cytoskeleton [42] and it has been found only in post mitotic neural cells during chicken embryo development [43], [44] thus suggesting an important role in brain morphogenesis. In our study, we found decreased phosphorylation of MARCKS at three different sites: Ser27, Ser138 and Thr143 in Cdk5^−/−^ brain. Interestingly, it was previously suggested that Ser27 (or Ser25 in chicken) could be phosphorylated by Cdk5 [25] and the bioinformatics analyses using a Cdk5 specific PSSM [5]. The NetphosK [31] tools confirmed that Ser27 had the higher probability to be phosphorylated by Cdk5. To further confirm this Cdk5-mediated phosphorylation at Ser27 site in MARCKS, we overexpressed chicken MARCKS and p35 into N2A cells and then evaluated Cdk5-dependent phosphorylation of Ser25 using an specific antibody [25]. Our results clearly indicated that phosphorylation of Ser25 was increased in N2A cells over-expressing p35 and it was decreased with the treatment with roscovitine of N2A cells and rat hippocampal neurons, suggesting that the phosphorylation of this residue is dependent of the Cdk5 activity. Additionally, MARCKS phosphorylation by PKC is regulated by PP2A, PP1 and PP2B [25]. We hypothesize that the Cdk5-dependent phosphorylation of mouse MARCKS at Ser27 can positively regulate the binding of MARCKS to the plasma membrane and actin filaments. In this context, it was reported that the non-PKC phosphorylated MARCKS (membrane-bound) is involved in memory formation and contributes to stabilization of synaptic morphology [45]. Because Cdk5 is also involved in learning and memory processes [46], [47] we speculate that Cdk5 plays an important role in these processes through phosphorylation of MARCKS at Ser27 (Figure 4A).

We also found that Grin1 displayed decreased phosphorylation in two residues Ser369 and Ser691 in Cdk5^−/−^ brain. Ser369 corresponds to Cdk5 SPXK motif [5] whereas Ser691 corresponds to the Cdk5 KSP motif [34]. Moreover, Grin1 has two more sites which include a minimal consensus motif for Cdk5 phosphorylation, Ser519 and Ser622. Although Ser519 and Ser622 sites in Grin1 were previously reported to be phosphorylated in brain [48], [49], our phosphoproteomic analysis found significant decrease only in the phosphorylation of Ser369 and Ser691 sites. This suggests that the phosphorylation of Ser519 and Ser622 could be dependent on other kinases. Since we used an antibody that specifically detects phosphorylation by Cdk5 on the SPXK motif, Ser369 is the epitope in Grin1 phosphorylated by Cdk5. When we overexpressed p35 in N2a cells, we observed a significant increase in the serine phosphorylation of Grin1. This was recognized by the same antibody, while roscovitine treatment restored phosphorylation to the basal level. This indicated that phosphorylation of Ser369 on Grin1 is dependent on the Cdk5 kinase activity Grin1, Gap43 and Gα_i/o_ protein are part of a G-couple receptor signaling pathway that regulates neurite growth in neural cells [50]. Interestingly, Gap43 is another protein which is differentially phosphorylated in Cdk5 null brains (Table 1). Grin1 does not contain conserved protein-protein interaction domains, however, it was reported its interaction with the activated subunits of G_z_/G_i_ and G_o_ [51] which are the proteins associated with G protein coupled receptors (GPCRs). Grin1 is located mainly at neuronal growth cones and when it is co-expressed with G_o_ in N2A cells induces neurite elongation, suggesting that Grin1 is an effector of G_o_ [52]. Besides, the co-expression of constitutively active G_o_ and Grin1 are related to increase Cdc42 activity [17]. It was reported that Cdk5/p35 complex have been associated with motility and stabilization of growth cone during the axon elongation [53], [54]. Our results suggest that the phosphorylation of Ser369 on Grin1 could be part of a network signaling controlled by Cdk5, regulating the elongation and maintenance of axons as well as the stability of growth cones. The stimulation of some GPCRs caused MAPK cascade activation [55]. Also, the signal transduction activated by second messenger-dependent kinases and the crosstalk between GPCRs and tyrosine kinases can induce ERK1/2 activation [56]. Interestingly, the ERK1/2 signaling pathway is a major regulator of Cdk5 activity through control of Egr1 and p35 expression [57]–[59]. Therefore, we suggest that extracellular signals that stimulate GCPRs with a subsequent activation of ERK1/2 can induce the expression of p35 increasing Cdk5 activity and maintaining a sustained response in time, reinforcing a potential signaling cascade through Grin1 necessary for axonal growth (Figure 4B).

Amongst the candidates identified to be potential Cdk5 substrates there is a group of proteins involved in the regulation of microtubule dynamics. This group encompasses Collapsin response mediator protein 1 (crmp1), Collapsin response mediator protein 2 (crmp2), Collapsin response mediator protein 4 (crmp4), microtubule-associated protein 1B (MAP1B), microtubule associated protein 2 (MAP2), tau, doublecortin (DCX) and stathmin. It has been previously shown that crmp1, crmp2 and crmp4 are phosphorylated by Cdk5 [60]–[62]. MAP1B is the first MAP expressed during nervous system development [63]–[64]. When phosphorylated by proline-directed protein kinases, such as gsk3β [65], JNK [66] and Cdk5 [67], becomes highly enriched in the axonal compartment. Currently; antibodies directed against phosphorylated MAP1B are insensitive to Cdk5 inactivation. Therefore, it is likely that epitopes found differentially phosphorylated in this study may serve to identify novel MAP1B phosphorylation involved in axon formation. DCX is a microtubule-associated protein involved in neuronal migration [68]. Phosphorylation of Ser297 in DCX is mediated by Cdk5 and regulates neuronal migration [69]. However, other DCX phosphoepitopes had been described including Ser339 found in this study [70]. It will be interesting to address the consequences for Thr336 and Ser339 phosphorylation upon microtubule dynamics and neuronal migration, the canonical DCX functions. MAP2 is a novel potential Cdk5 substrate. Previously, it was shown that MAP2 can be phosphorylated in CAD cells displaying increased Cdk5 activity [71]. However, the functional role for Cdk5-dependent MAP2 phosphorylation still remains elusive. It is tempting to speculate that such phosphorylation may be related with changes in dendrite formation and plasticity. Tau, is an axonal microtubule associated protein widely expressed in the nervous system. It is abnormally phosphorylated in brain of patients with Alzheimer's disease [72]. Amyloid-β peptide induce tau phosphorylation by activating protein kinases such as gsk3β [73] and Cdk5 [74]. Therefore, decreased tau phosphoepitopes here presented may serve as molecular markers for neurodegeneration associated to Cdk5 functions.

In summary, our phosphoproteomics analysis of Cdk5 null brain identified decreased phosphorylation in several potential Cdk5 proteins that are involved in neuronal morphology, metabolism and signal transduction. These phosphoproteome data may provide a basis for identifying new Cdk5 substrates; however, further work is required to determine how the novel substrates or sites identified in our study regulates process such as cytoskeleton dynamics, neuronal migration and synapses formation and stability.

## Supporting Information

Table S1 (PDF)
