# Supplementary material for: Searching for Novel Cdk5 Substrates in Brain by Comparative Phosphoproteomics of Wild Type and Cdk5−/− Mice
Source: PLoS One. 2014 Mar 21;9(3):e90363. doi: 10.1371/journal.pone.0090363 (PMC3962345; doi:10.1371/journal.pone.0090363)
Supplement: Table S1 — (PDF) [file pone.0090363.s001.pdf]

Protein	Gene	Localization	Technique	PMID
40S ribosomal protein S3	Rps3	Pyramidal cells of hypocampus and dentate gyrus of adult brain	IHC	22274408
Abl interactor 1	Abi1	Synaptosomes of adult brain and growth cones of neonatal brain	WB	10995551
Alpha-adducin	Add1	Dendritic spines of cultured hyppocampal neurons	ICC	9679146
APC membrane recruitment protein 2	Amer2	Strongly expressed in neurons of central and peripheral nervous system	ISH	20503382
Bcl-2-associated transcription factor 1	Bclaf1			N/A
Calmodulin-regulated spectrin-associated protein 1	Camsap1	Microtubule-associated pattern in cerebelar granule cells. Not evaluated in other brain cells	ICC	19508979
Calmodulin-regulated spectrin-associated protein 3	Camsap3			N/A
Clathrin coat assembly protein AP180	Snap91	Clathrin-coated vesicles and plasma membrane of synaptic compartments	EM	18842885
Collapsin response mediator protein 1	Dpysl1	Soma, dendrites and axons of embryonic cortical neurons	ICC	22378692
Collapsin response mediator protein 2	Dpysl2	Soma, dendrites and axons of embryonic cortical neurons	ICC	22378692
Collapsin response mediator protein 4	Dpysl3	Soma, dendrites and axons of embryonic cortical neurons	ICC	12745088
DNA ligase 1	Lig1			N/A
E3 ubiquitin-protein ligase TRIM2	Trim2	Soma and neurites of unpolarizated hippocampal neurons. Excluded for axon in madure neurons	ICC	20796172
G protein-regulated inducer of neurite outgrowth 1	Gprin1	Highly enriched in growth cones	WB, ICC	10480904
Growth-associated protein 43	Gap43	Neuron-specific expression. Highly enriched in growth cones and synaptosomal plasma membranes	IHC	441300
Hepatoma-derived growth factor	Hdgf	Mainly expressed in neurons and localized in nucleus	IHC	15140875
Heterogeneous nuclear ribonucleoprotein D0	Hnrnpd	Purkinje and granular cells of postnatal cerebellum. Not determined in other areas.	IHC	19115409
Kinesin light chain 1	Klc1	Axon of hippocampal neurons	ICC	22582169
MARCKS-related protein	Marcksl1	Hippocampal and cortical neurons in culture	WB	22751924
Microtubule-associated protein 1B	Map1b	Neuron-specific expression. Present in soma, dendrites and axons	IHC	2562784
Microtubule-associated protein 2	Map2	Highly expressed in differenciated neurons	ICC	7001466
Microtubule-associated protein tau	Mapt	Neuron-specific expression. Axonal distribution.	IHC	3930508

Myristoylated alanine-rich C-kinase substrate	Marcks	Small dendrites, axon and axon terminals	IHC, EM	2332803
Na(+)/H(+) exchange regulatory cofactor NHE-RF1	Slc9a3r1	No evidence of neuronal expression. Weak expression in reactive astrocyes	IHC	19308292
Nestin	Nes	Neuronal precursor cells	ICC	2172829
Neuronal migration protein doublecortin	Dcx	Widely expressed in migrating neurons. Microtubule-asociatted pattern	IHC, ICC	10399933
Phosphatidylinositol 4-kinase beta	Pi4kb	Perycaria and dendrites of hippocampal neurons. Not present in axons.	ICC, EM	12244129
Plakophilin-4	Pkp4	Protrusions of astrocytes	WB, ICC	21858875
Programmed cell death protein 4	Pdcd4	Expressed in primary cortical neurons and developing cortex	WB	22757755
Protein SDE2 homolog	Sde2			N/A
Ras GTPase-activating protein-binding protein 1	G3bp1	Large granular structures in soma of hippocampal neurons and Purkinje cells	ICC, IHC	23373770
Ras GTPase-activating protein-binding protein 2	G3bp2			N/A
Rho GTPase-activating protein 1	Arhgap1			N/A
RNA-binding protein Raly	Raly			N/A
SAFB-like transcription modulator	Sltm			N/A
Serine/threonine-protein kinase DCLK1	Dclk1	Radially migrating neurons in cerebral cortex	IHC, ICC	11124993
Stathmin	Stmn1	Predominantly in soma and dendrites of cultures cortical neurons	ICC	9452014
Stathmin-2	Scg10	Neuron-specific expression. Highly concentrated in growth cones of developing neurons	ICC	3272176
T-box brain protein 1	Tbr1	Highly expressed in the nucleus of developing neurons. Synaptosomal localization in postnatal neurons	IHC	17329080
U1 small nuclear ribonucleoprotein 70	Snrnp70			N/A

This Supplementary Table summarizes experimental evidences supporting neuronal distribution of putative Cdk5 new substrates identified in this study.
The table shows the article where substrate was shown to be present in neurons (PMID column) and methodologies employed (Technique)

Grey cells indicate candidates experimentally validated in the present study
